# Supplementary material for: Osteotropic Effect of Parenteral Obesity in Programmed Male Rats Fed a Calorically Differentiated Diet during Growth and Development
Source: Animals (Basel). 2022 Sep 6;12(18):2314. doi: 10.3390/ani12182314 (PMC9495023; doi:10.3390/ani12182314)
Supplement: Supplementary file 1 [file animals-12-02314-s001.zip › Table S1.pdf]

Table S1. The content of the standard (S) and high-energy (F) diets.

Ingredients	Standard diet (S)	High-energy diet (F)
Metabolic Energy	11.50 MJ/kg	17.6 MJ/kg
Crude protein	16.00%	19.0%
Crude fat min.	2.80%	16.0%
Crude ash max.	7.00%	5.58%
Crude fiber max.	5.00%	3.86%
Carbohydrate	65%	65%
Lysine	0.80%	1.2%
Methionine + Cysteine	0.50%	0.76%
Tryptophan	0.190%	0.27%
Vitamin A	8000 IU/kg	8000 IU/kg
Vitamin D3	1000 IU/kg	1000 IU/kg
Vitamin E	50 mg/kg	76.9 mg/kg
Vitamin K3	0.24%	0.24%
Vitamin B1	0.8%	0.8%
Vitamin B2	0.6%	0.6%
Vitamin B6	1.1%	1.1%
Vitamin B12	0.004%	0.004%
Biotin	0.04%	0.04%
Folic acid	0.2%	0.2%
Nicotinic acid	7.78%	7.78%
Pantothenic acid	2.17%	2.17%
Linoleic acid	2%	2%
Calcium	1.10 %	1.32%
Phosphorus	0.70 %	0.64%
Natrium	0.22 %	0.19%
Magnezium	0.3%	0.3%
Manganese	0.005%	0.005%
Iodine	0.02%	0.02%
Copper	1.16%	1.16%
Sulfur	0.14%	0.14%
Zinc	6.26%	6.26%
Selenium	0.03%	0.03%
