# Supplementary figures and images for: Osteotropic Effect of Parenteral Obesity in Programmed Male Rats Fed a Calorically Differentiated Diet during Growth and Development
Source: Animals (Basel). 2022 Sep 6;12(18):2314. doi: 10.3390/ani12182314 (PMC9495023; doi:10.3390/ani12182314)

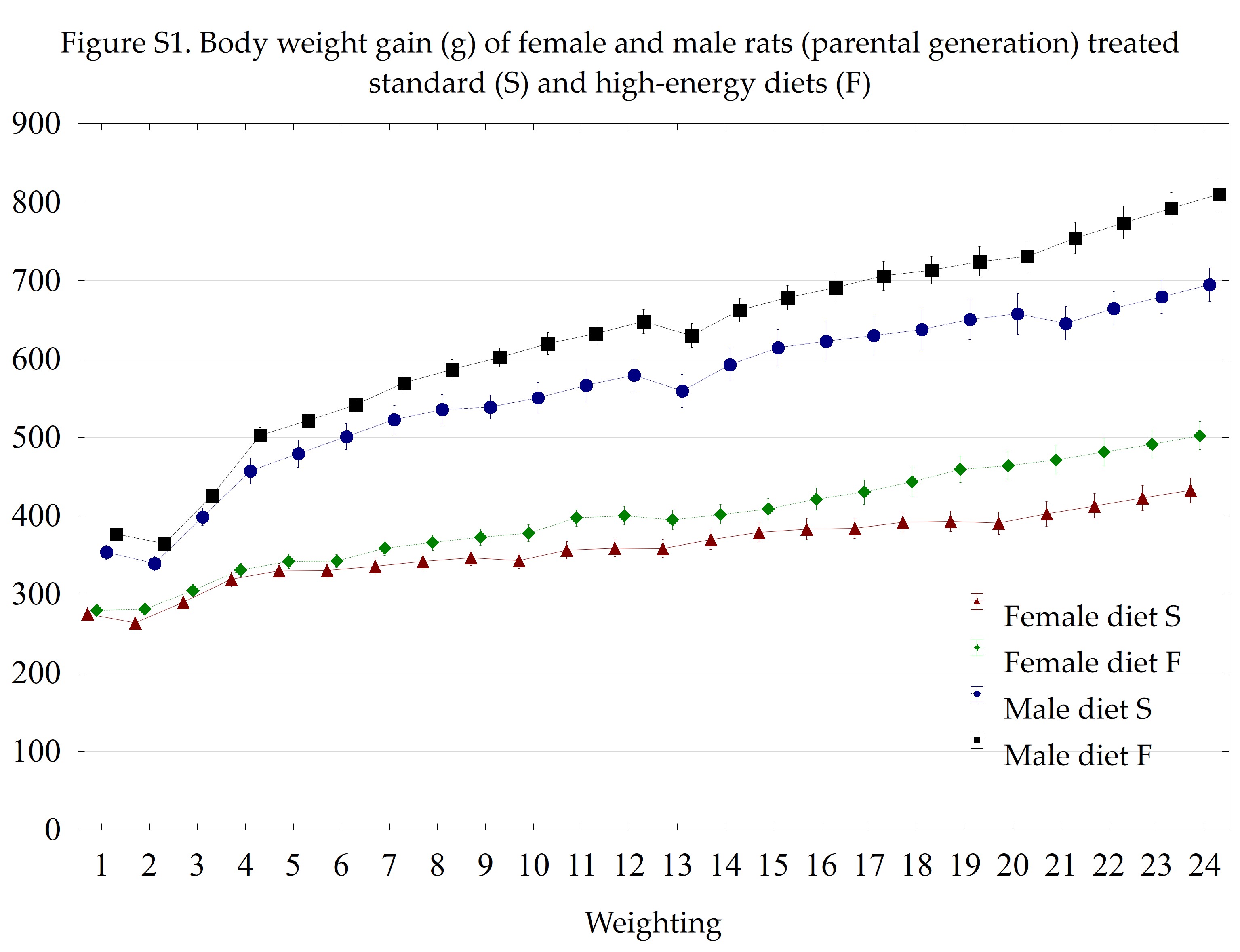

Supplement: Supplementary file 1 [file animals-12-02314-s001.zip › Figure_S1.jpg]
